# Trends in smoking during pregnancy stratified by the use of opioid agonist therapy and the contribution of smoking to poor outcome in neonates prenatally exposed to opioid agonist treatment

**DOI:** 10.1007/s00737-023-01342-z

**Published:** 2023-06-27

**Authors:** Erin Kelty, Alys Havard, David B. Preen

**Affiliations:** 1 The School of Population and Global Health, The University of Western Australia, 35 Stirling Highway, Nedlands, Western Australia Australia; 2 National Drug and Alcohol Research Centre, UNSW Sydney, Sydney, Australia; 3 School of Population Health, UNSW Sydney, Sydney, Australia

**Keywords:** Buprenorphine, Cigarette smoking, Methadone, Opioid dependence, Opioid use disorder, Pregnancy

## Abstract

High rates of cigarette smoking have been observed in pregnant women on opioid agonist therapy (OAT). However, it is unclear if these rates have changed overtime in line with the general population and the degree to which smoking contributes to poor outcomes in neonates born to women on OAT. Women who gave birth in Western Australia (WA) between 2003 and 2018 were identified from whole-population midwives records. Linked records were used to identify women who had been dispensed OAT during pregnancy and those who had smoking during pregnancy. Temporal changes in smoking during pregnancy were examined for women on OAT (*n* = 1059) and women not on OAT (*n* = 397,175) using Joinpoint regression. In women treated with OAT during pregnancy, neonatal outcomes were compared between smoking and non-smoking women using generalised linear models. During the study period, 76.3% of women on OAT smoked during pregnancy compared with 12.0% of the general population. There was a decrease in the prevalence of smoking during pregnancy among women not on OAT (APC: − 5.7, 95%CI: − 6.3, − 5.2), but not in women on OAT (APC: 0.8, 95%CI: − 0.4, 2.1). For women receiving OAT, smoking was associated with an increased odds of low birth weight (OR: 1.57, 95%CI: 1.06, 2.32) and neonatal abstinence syndrome (OR: 1.34, 95%CI: 1.01, 1.78) compared with non-smoking. Despite reductions in the prevalence of smoking during pregnancy in the general population, similar reductions have not occurred in pregnant women on OAT. The high prevalence of smoking in pregnant women on OAT is contributing to poor neonatal outcomes.

**Supplementary Information:**

The online version contains supplementary material available at 10.1007/s00737-023-01342-z.

## Introduction

Cigarette smoking during pregnancy is associated with a range of poor maternal and neonatal health outcomes including miscarriage, low birth weight, stillbirth, and pre-term birth (Pineles et al. 2014; Marufu et al. 2015; Abraham et al. 2017; Avşar et al. 2021). Globally the prevalence of smoking in pregnancy is estimated to be 1.7% (95% CI 0.0–4.5%) based on studies published between 1985 and 2016, with substantial variation between regions (Lange et al. 2018). More recent estimates in high-income countries have reported higher rates of smoking, including 6.9% in the USA (2017) (Azagba et al. 2020), 9.0% in Denmark (2017) (de Wolff et al. 2019), 9.3% in Australia (2019) (Anon 2022), and 10.9% in Germany (2010–2016) (Kuntz et al. 2018). Additionally, within countries there is substantial difference in rates of smoking in pregnancy associated with factors. Protective factors include high socio-economic status, higher education ascertainment, and being married, while risk factors include teenage pregnancies, mental health disorders, First Nations people, and living in rural areas (Mohsin et al. 2011; Ekblad et al. 2013; Goodwin et al. 2017; Azagba et al. 2020). In line with trends in the general population, decreases in smoking in pregnancy have been observed in numerous countries including Australia (Havard et al. 2018), Denmark, Norway, Sweden (Ekblad et al. 2013), and the USA (Li et al. 2018). However, there is evidence to suggest that reductions in the prevalence of smoking in pregnancy may not be occurring consistently across all demographics, with the largest reduction observed in women of a high socio-economic status, while only small reductions were observed in teen mothers and women from remote areas (Mohsin et al. 2011). In some populations, increases in smoking during pregnancy have been observed, for example, in women diagnosed with depression (Goodwin et al. 2017).

The use of illicit opioids is associated with high prevalence of smoking, both in the general population and in pregnant women (Harrell et al. 2012; Chisolm et al. 2013). Despite the high prevalence and poor associated outcome, smoking cessation is typically not addressed in pregnant women with an OUD, possibly due to the often complex nature of these patients. The co-use of tobacco and opioids is of concern because of the potential additive risk of adverse maternal and foetal outcomes (Oga et al. 2018). The high prevalence of smoking in pregnant illicit opioid users is also reflected in pregnant women on opioid agonist therapy (OAT) (typically methadone or buprenorphine) for the treatment of opioid use disorder (OUD) (Chisolm et al. 2013; Meyer et al. 2015; Kelty and Hulse 2017a). For example, in an Australian study of children born between 2001 and 2011, the percentage of women who smoked during pregnancy was 76.5% for women on methadone, 75.8% for women on buprenorphine, and 13.0% for women without an OUD (Kelty and Hulse 2017b). The use of methadone or buprenorphine during pregnancy is associated with poor neonatal outcomes compared with the general population (Kelty and Hulse 2017a), which may be compounded by tobacco use. For example, smoking during pregnancy in women receiving OAT has been shown to be associated with more severe symptoms in neonates with neonatal abstinence syndrome (NAS) (Choo et al. 2004; Jones et al. 2013). Reducing smoking in pregnant women on OAT has the potential to reduce the risk of poor maternal and neonatal outcomes. However, it is currently unclear if smoking during pregnancy in women on OAT has reduced in recent years, as per the wider population of pregnant women.

This study examines changes in the rates of smoking among pregnant women stratified by their use of OAT, between 2003 and 2018 in Western Australia (WA). Additionally, the study examines the relationship between smoking and adverse outcomes in neonates exposed to OAT in utero.

## Methods

This study comprised a retrospective whole population cohort of women who gave birth in WA between 2003 and 2018. These women were identified from the WA Midwives Notification System. This statutory dataset contains information collected at the time of birth, including self-reported smoking status during pregnancy, for all live and stillborn neonates of at least 20 weeks gestation (or 400 g birthweight if gestation is unknown) born in WA. For a subset of neonates (born 2010–2018), data was available on the average number of cigarettes smoked per day prior to 20-week gestation and from 20-week gestation onwards. Data from the Midwives Notification Scheme were linked with the WA Monitoring of Drugs of Dependence System to identify women who were treated with OAT during pregnancy. The Monitoring of Drugs of Dependence System records the dispensing of all schedule 8 medications (medications with a recognised therapeutic need but a higher risk of misuse, abuse, and dependence and are therefore subject to restrictions in terms of prescribing and dispensing) in WA. Within this system, a distinction is made between medications for the treatment of opioid use disorders (methadone, buprenorphine, and combined buprenorphine/naloxone) and other schedule 8 medications including opioids for the treatment of pain.

The proportion of women who smoked during pregnancy was calculated for each year based on the year in which the neonate was born (2003–2018), stratified by their use of OAT (yes/no). Women were classified as having received OAT during pregnancy if they had been dispensed one or more prescription for OAT during pregnancy. Within the MODDS data, dispensing was provided as month/year (no day); thus, dispensing during pregnancy was defined as being dispensed methadone or buprenorphine in the month of conception through to the month prior to birth. Pregnancy dates were estimated based on the date of birth and the estimated length of gestation available in the Midwives Notification System. This is the gestation estimated by the midwife at time of birth with reference to the LMP, EDD, and appearance of infant. Joinpoint regression (otherwise known as segmented regression or change point regression) performed using the Joinpoint Regression Program (version 4.8.0.1) was used to examine trends over time. In brief, Joinpoint regression is a method of analysis that examines trends over time but allows for trends to change by forming a number of segments. For each segment, the annual percentage change (APC) is calculated and tested for significance using the Monte Carlo permutation method. A number of models are created with varying numbers of segments/joinpoints (starting with 1 segment/0 joinpoints), and the most appropriate model is selected. Comparisons in the prevalence of smoking between women dispensed OAT during pregnancy and those who were not were performed using univariable logistic regression. Neonatal outcomes were compared for women on OAT who had smoked and not smoked during pregnancy. Outcomes examined included perinatal mortality (stillbirth and death within 28 days of birth), estimated length of gestation, pre-term birth, birth weight, low birth weight (< 2500g), NAS, length of stay, and admission to the special care nursery. These outcomes were identified from the Midwives Notification System, the Hospital Morbidity Data Collection, and the WA Death Register. Comparison between smokers and non-smokers was made using univariable generalised linear models and multivariable models adjusting for maternal age, prior pregnancies (yes/no), and low socio-economic status. Maternal age, prior pregnancies, and low socio-economic status were obtained from the MNS, with low socio-economic status was derived from the address provided at the time of birth. Socioeconomic status was inferred from the socio-economic indexes for areas (SEIFA) using the index of relative socio-economic disadvantage calculated by the Australian Bureau of Statistics (ABS 2013) and classifying low socio-economic status as the lowest 20%.

The study was approved by the WA Department of Health Human Research Ethics Committee (RGS0000003029) and the University of Western Australia Human Research Ethics Committee (RA/4/20/5530). To protect patient confidentiality, the data utilized in this study is not publically available. This is a condition of data access. Code used in the study is available from the corresponding author upon reasonable request.

## Results

**Fig. 1:**
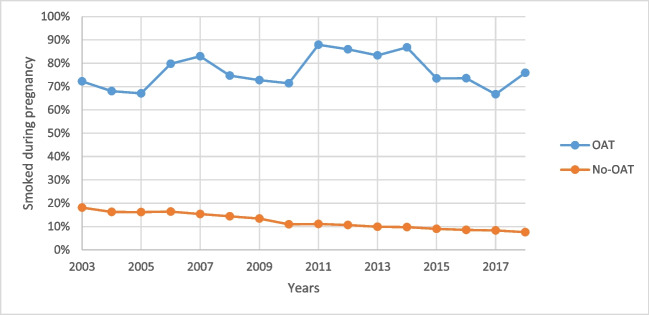
The percentage of women who smoked during pregnancy by birth year, stratified by the use of opioid agonist therapy (OAT)

Between 2003 and 2018, 398,234 women gave birth to live or stillborn neonates of at least 20-week gestation (or > 400 g birth weight if gestation was unknown) in WA. Of these women, 1059 (0.3%) were treated with methadone (*n* = 707, 66.8%) and/or buprenorphine (*n* = 465, 43.9%) during pregnancy (113 treated with both). In women on OAT, 76.3% (*n* = 808) smoked during pregnancy, in comparison, for women not on OAT, 12.0% (*n* = 47,629) of women smoked during pregnancy (OR: 23.62, 95%CI: 20.50, 27.23) (Fig. 1). Women on OAT were also more likely to have previously been pregnant and live in an area of low socio-economic status (Table 1).

**Table 1:** The characteristics of women at birth, stratified by opioid agonist treatment (OAT) dispensing during pregnancy.

	OAT	No OAT
Number	1059	397,175
Smoked during pregnancy, *n* (%)	808 (76.3%)	47,629 (12.0%)
Age (years), mean ± sd	30.1 ± 5.1	30.4 ± 5.6
Previous pregnancies, median (IQR)	3 (1, 4)	1 (0, 2)
Low socio-economic status^1^, *n* (%)	168 (15.7%)	32,345 (8.0%)
Residing in regional/remote area, *n* (%)	56 (5.3%)	33,468 (8.4%)

*N*, number; *IQR*, interquartile range; *OAT*, opioid agonist treatment

^1^Residing in a low socio-economic area (lowest 20%) at the time of birth, calculated based on Socio-Economic Indexes for Area (SEIFA)

For a subset of 370 women on OAT who smoked during pregnancy, the median number of cigarettes smoked on average per day was 10 (interquartile range (IQR): 8, 15) prior to 20 weeks of pregnancy, and 10 (IQR: 5, 15) from 20 weeks onwards. For the equivalent subset of women not on OAT (*n* = 21,725), the median number of cigarettes smoked per day was also 10 (IQR: 5, 12), but reduced to 5 (IQR: 2, 10) from 20 weeks onward.

During the study period, there was a significant decrease in the percentage of smoking during pregnancy among women not on OAT (APC: − 5.7, 95%CI: − 6.3, − 5.2). In women on OAT, there was no significant change in the percentage smoking during pregnancy (APC: 0.8, 95%CI: − 0.4, 2.1) (Supplementary Fig. 1).

A total of 1069 neonates were born to the 1059 women treated with OAT. In neonates exposed to OAT in utero, smoking was associated with a reduction in average birth weight of 154 g and an 57% increased odds in neonates being classified as low birth weight (< 2500 g) compared with neonates born to women on OAT who did not smoke during pregnancy (Table 2). Smoking was associated with a 34% increased odds of being diagnosed with NAS compared with neonates born to women on OAT who did not smoke during pregnancy. Despite this, smoking was not associated with an increased risk of admission to the special care unit, increased length of hospital stay, or an increase in perinatal mortality.

**Table 2:** The association between smoking status and neonatal outcomes in neonates exposed to opioid agonist therapy in utero

	Non-smoking	Smoking	Crude effect estimate (95%CI)	Adjusted effect estimate (95%CI)^1^
Number	255	814	-	-
Perinatal mortality, *n* (%)	8 (3.1%)	19 (2.3%)	OR: 0.74 (0.32, 1.71)	OR: 0.68 (0.29, 1.59)
Estimates gestation (weeks), mean ± sd	37.8 ± 2.9	37.5 ± 2.8	Coef: − 0.23 (− 0.63, 0.17)	Coef: − 0.17 (− 0.58, 0.23)
Pre-term birth, *n* (%)	45 (17.7%)	153 (18.8%)	OR: 1.08 (0.75, 1.56)	OR: 1.06 (0.73, 1.53)
Birth weight (g), mean ± sd	3031 ± 698	2900 ± 629	Coef: − 131 (− 222, − 40)	Coef: − 123 (− 214, − 31)
Low birth weight (< 2500), *n* (%)	36 (14.1%)	167 (20.5%)	OR: 1.57 (1.06, 2.32)	OR: 1.54 (1.04, 2.28)
Length of stay (days), median (IQR)	6 (4, 11)	6 (4, 13)	IRR: 1.07 (0.94, 1.23)	IRR: 1.08 (0.94, 1.24)
Neonatal abstinence syndrome, *n* (%)	127 (49.8%)	465 (57.1%)	OR: 1.34 (1.01, 1.78)	OR: 1.33 (1.00, 1.77)
Admission to special care nursery, *n* (%)	116 (46.4%)	357 (44.7%)	OR: 0.94 (0.70, 1.24)	OR: 0.93 (0.70, 1.25)

^1^Adjusted for maternal age, previous pregnancy (yes/no), and residing in a low socio-economic area

## Discussion

Despite continual reductions in the percentage of women smoking in pregnancy within the general population over the last couple of decades (Ekblad et al. 2013; Havard et al. 2018; Li et al. 2018), there was no such decline in women on OAT observed between 2003 and 2018 in WA, resulting in a growing disparity between the two groups. Consistent with our findings, Weinberger et al. (2018) observed no change in smoking prevalence in persons with a substance use disorder in the USA between 2002 and 2016 (Weinberger et al. 2018). When cannabis use disorders were excluded, the researchers observed a significant increase in smoking. Increases in rates of smoking during pregnancy have also been observed in women diagnosed with depression (Goodwin et al. 2017), which is a common comorbidity in women with OUD (Peles et al. 2007; Arnaudo et al. 2017).

Interestingly, while the number of cigarettes smoked during pregnancy fell in women not on OAT, there was no change in women on OAT. This may reflect additional barriers to stopping or reducing smoking in women on OAT, including the pro-smoking social norms and a lack of evidence-based smoking cessation medication suitable for use in pregnancy and in combination with OAT (Vlad et al. 2020). Additionally, clinicians may view smoking low priority in comparison to other health behaviours such as intravenous drug use.

In neonates exposed to OAT in utero, smoking was associated with a reduction in average birth weight and a corresponding increase in the percentage of neonates classified as being of low birth weight. Smoking during pregnancy has been consistently associated with reductions in birth weight in the general population (Abraham et al. 2017), although the mechanism by which this occurs is not fully understood (Suter and Aagaard 2020).

Smoking during pregnancy was also associated with a 34% increase in the odds of a neonate being diagnosed with NAS in our study. The association between smoking and NAS has been reported elsewhere (O'Donnell et al. 2009; Kaltenbach et al. 2012; Desai et al. 2015). However, a number of studies have also found no association (Seligman et al. 2008; Liu et al. 2010; Cleary et al. 2011). The possible association between smoking and NAS may be in part attributable to neonatal nicotine withdrawal syndrome, as both present with similar symptoms (GarcÍA-Algar et al. 2001). However, the association may alternatively due to unmeasured confounding.

Interestingly, cigarette smoking was not significantly associated with other neonatal outcomes including length of gestation or length of hospital stay. While both smoking and OAT have been associated with both outcomes, the combination did not appear to have an additive affect (Kalien 2001; Kelty and Hulse 2017b). The association between smoking and outcomes in neonates exposed to OAT in utero should be cautiously interpreted given the potential for confounding by other factors and thus may not be causal. For example, women who choose not to smoke during pregnancy may be more likely to refrain from other risky behaviours such as alcohol consumption that have been shown to contribute to poor neonatal outcomes (Chiolero et al. 2006).

Traditionally, it was assumed that pregnant women on OAT would be reluctant to stop or reduce their use of cigarettes or other drugs. However, pregnancy is often a period of substantial motivation for positive change, and pregnant women on OAT may be willing to consider reducing or stopping smoking (Anon 2018). While smoking cessation may not be a realistic goal for all women on OAT, providing support for women who are receptive could have substantial health benefits for both the mother and her child.

### Conclusion

While smoking during pregnancy has declined in WA between 2003 and 2018, no changes were observed in women on OAT. Reducing smoking in women on OAT could result in improved neonatal outcomes, including increases in birth weight and reductions in NAS.

## Supplementary information

ESM 1 Supplementary Figure 1: Joinpoint regression fitted to the percentage of women who smoked during pregnancy for women not on OAT (top) and on OAT (bottom). (DOCX 165 kb)
