# Supplementary figures and images for: Trends in smoking during pregnancy stratified by the use of opioid agonist therapy and the contribution of smoking to poor outcome in neonates prenatally exposed to opioid agonist treatment
Source: Arch Womens Ment Health. 2023 Jun 27;26(4):543–8. doi: 10.1007/s00737-023-01342-z (PMC10333411; doi:10.1007/s00737-023-01342-z)

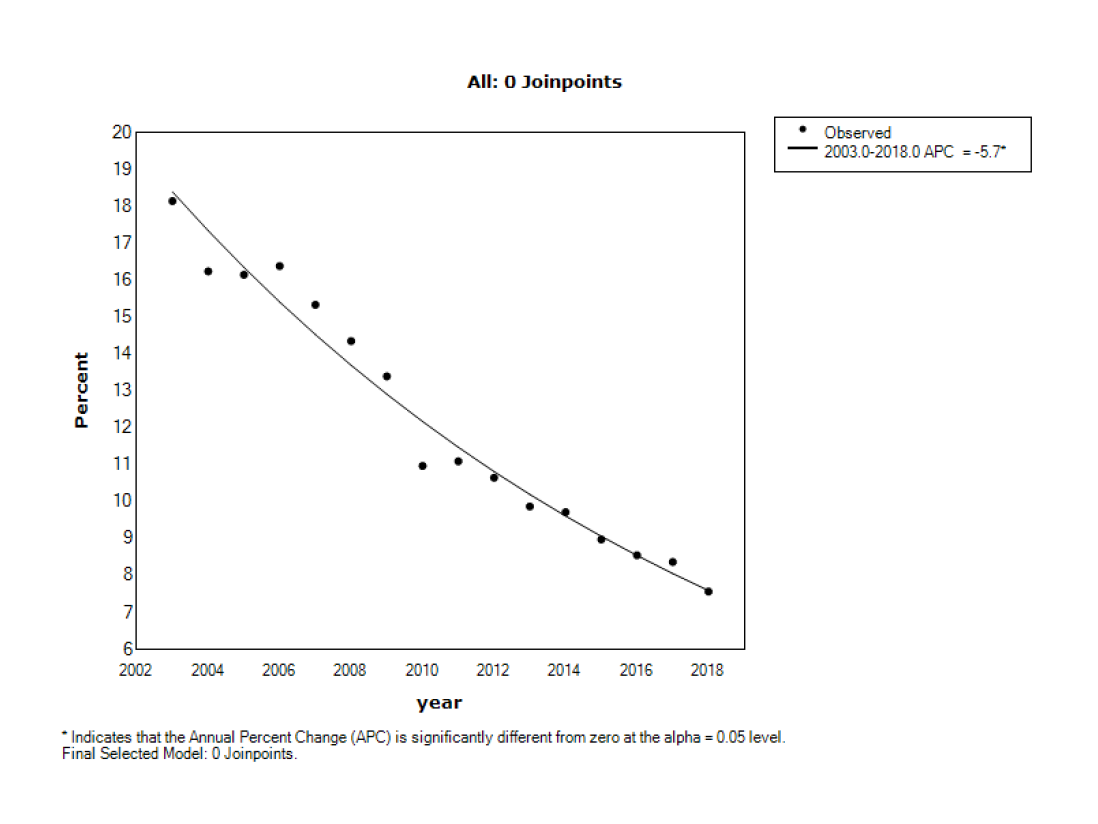


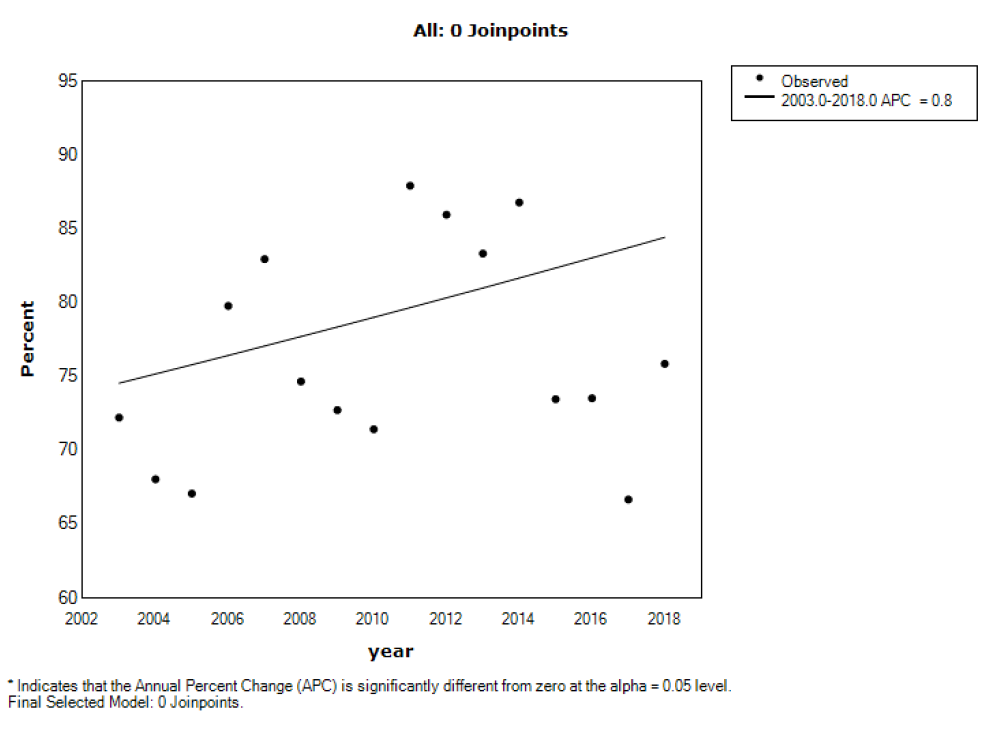

Supplement: Supplementary file 1 — Supplementary Figure 1: Joinpoint regression fitted to the percentage of women who smoked during pregnancy for women not on OAT (top) and on OAT (bottom). (DOCX 165 kb) [file 737_2023_1342_MOESM1_ESM.docx]
